# Multi-Objective Sliding Mode Control on Vehicle Cornering Stability with Variable Gear Ratio Actuator-Based Active Front Steering Systems

**DOI:** 10.3390/s17010049

**Published:** 2016-12-28

**Authors:** Xinbo Ma, Pak Kin Wong, Jing Zhao, Zhengchao Xie

**Affiliations:** 1 Department of Electromechanical Engineering, University of Macau, Taipa, Macau 999078, China; yb57467@umac.mo (X.M.); fstpkw@umac.mo (P.K.W.); 2 School of Mechanical and Automotive Engineering, South China University of Technology, Guangzhou 510641, China; zxie001@yahoo.com

**Keywords:** cornering stability, AFS system, VGRS actuator, SMC strategy

## Abstract

Active front steering (AFS) is an emerging technology to improve the vehicle cornering stability by introducing an additional small steering angle to the driver’s input. This paper proposes an AFS system with a variable gear ratio steering (VGRS) actuator which is controlled by using the sliding mode control (SMC) strategy to improve the cornering stability of vehicles. In the design of an AFS system, different sensors are considered to measure the vehicle state, and the mechanism of the AFS system is also modelled in detail. Moreover, in order to improve the cornering stability of vehicles, two dependent objectives, namely sideslip angle and yaw rate, are considered together in the design of SMC strategy. By evaluating the cornering performance, Sine with Dwell and accident avoidance tests are conducted, and the simulation results indicate that the proposed SMC strategy is capable of improving the cornering stability of vehicles in practice.

## 1. Introduction

In recent decades, cornering stability has been an inevitable topic in terms of the vehicle performance, and the cornering performance directly influences the handling stability and vehicle active safety [1]. As the main property of lateral dynamics, vehicle cornering stability is greatly influenced by the steering system. In the past several decades, the steering system has been developed through five stages, including the mechanical steering stage, hydraulic steering stage, electro-hydraulic steering stage, electric power steering, and active front steering (AFS) stage [2]. In the five stages, the AFS system becomes attractive with the advantages in enhancing cornering dynamics by providing (1) steering comfort by reducing driver’s steering effort; and (2) active safety, as well as cornering stability [3]. Though the AFS system will lead to intrusiveness in the driver’s action, it can bring advantages to inexperienced and nonprofessional drivers. In view of the effectiveness of the AFS system, this work also aims to study the characteristics of the AFS system.

However, in the previous studies of AFS systems, most attention has been paid to improve the stability and agility by integrating the AFS system with other active systems, like anti-lock braking systems, traction control systems, anti-slip regulation, dynamic stability control, electronic stability programming, or direct yaw control [4,5,6]. These studies emphasized the coordination among different parts and came up with some corresponding control strategies, such as hierarchical control and multivariable control [7,8]. In other words, many investigations have been done in controller design for the integration of the AFS system with other active systems, but the control methods cannot fulfill the actual benefits of the AFS system completely. In light of the drawbacks of the existing studies, deep research on the AFS system, including design and control, is a desirable direction.

As for the design of the AFS system, two methods are commonly used to implement the function of the AFS system: (1) a mechanical AFS system with a variable steering gear ratio; and (2) a direct steer-by-wire (SBW) mechanism system without a direct mechanical linkage between the steering wheel and front wheel [9,10]. Although there are some studies on the SBW system, its safety and reliability cannot be guaranteed, especially when there is failure in the steering actuator. Hence, the mechanical AFS system is studied in this work.

The existing mechanical AFS systems have five kinds of actuators in practice: (1) a planetary gear set actuator with two mechanical inputs and a single mechanical output, which has been successfully applied to the BMW series [9,11]; (2) an adaptive steering actuator with a gear mechanism mounted right inside the steering wheel applied to Ford; (3) a dynamic steering actuator containing harmonic gear drive mechanism employed in Audi; (4) a direct steering actuator with a rack of variable tooth pitch used in Mercedes Benz; and (5) a variable gear ratio system (VGRS) actuator using a wave generator and a flexible gear which is employed in Toyota. They all have the advantages of stable working capacity and high efficiency. However, by comparing their mechanical structures with most actuators, the VGRS actuator being the most popular, reliable, and the fastest response design. Therefore, the AFS system with the VGRS actuator is studied in this work. Even though the VGRS actuator is available on the market, its mathematical model has not been available in the open literature yet. Thus, it is an original work to develop the mathematical model of VGRS.

As the core component of the AFS system, a controller plays a critical role in the improvement on cornering stability. In the previous investigations, various control approaches have been developed [4,5,7,8,12,13,14,15]. A predictive control model based on the online linearization of the vehicle model was proposed by Falcone to reduce the computational complexity [7]. In [8], an in-wheel-motored electric vehicle with an SBW system controlled by an inner-loop and outer-loop controller was proposed to guarantee the robust yaw stability [8]. In [14], a quantitative feedback theory was designed by incorporating the yaw rate sensor into the active steering system with considering the uncertain quantities. A single objective sliding mode control (SMC) was developed for the AFS system to improve the robustness [6]. However, most of them only consider the yaw rate to ensure the cornering stability, while the sideslip angle is neglected. Actually, the sideslip angle and yaw rate have influence on the cornering stability, because the sum of the sideslip angle and the yaw angle determines the dynamic state of the vehicle directly. When the sideslip angle is small, the dynamic state of the vehicle is determined by the yaw angle, which can be obtained by integrating the yaw rate with respect to time. When a severe sideslip occurs, the sideslip angle increases quickly and the yaw rate cannot describe the dynamic state of the vehicle accurately. Therefore, the sideslip angle and yaw rate should be considered together [4,6]. Thus, a multi-objective control strategy should be developed in this study. Considering that the SMC strategy performs well in dealing with two dependent objectives, a multi-objective SMC strategy is employed in this work with consideration of the sideslip angle and yaw rate together. Even though the application of SMC was available in [6], it was a single-objective control by purely regulating the yaw rate for achieving cornering stability. Therefore, the use of SMC for concurrent control of sideslip angle and yaw rate is an original work.

Based on the above discussion, an AFS system with the VGRS actuator is designed and controlled using a multi-objective SMC strategy so as to improve the cornering stability of vehicles. The main novelties of this paper can be summarized as follows: (1) in the design of the AFS system, different sensors are considered to measure the vehicle state, and the model of the AFS system with the VGRS actuator is developed and presented; and (2) in order to improve the cornering stability of vehicles, the two dependent objectives, including the sideslip angle and yaw rate, are considered together to design the multi-objective SMC strategy on the basis of a two-degrees-of-freedom (2-DOF) vehicle model. It is believed that this work can provide guidance for the design of the AFS system with the VGRS actuator and provide an alternative solution for control of dependent objectives. The rest of paper is organized as follows: Section 2 addresses the 2-DOF vehicle model. Section 3 presents the proposed AFS system with VGRS actuator. Section 4 provides the controller design for the proposed system. The simulation is presented in Section 5. Finally, conclusions of this work are given in Section 6.

## 2. 2-DOF Vehicle Model

**Figure 1:**
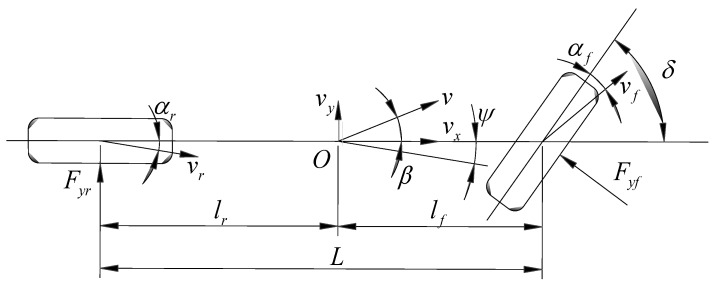
Two degrees-of-freedom (2-DOF) vehicle model.

Considering the lateral motion and yaw motion, the four-degrees-of-freedom (4-DOF) vehicle model and the 2-DOF vehicle model are quite popular plant or reference models in this field. The 4-DOF vehicle model is much more complicated than the 2-DOF vehicle model. Many factors should be considered in the 4-DOF model but they are not easily identified. Thus, this work considers the 2-DOF vehicle model which is developed to capture the main characteristics of the vehicle steering system as shown in Figure 1. Despite the reduced complexity, the 2-DOF vehicle model can still capture the relevant vehicle dynamics, and is appropriate for the design of SMC strategies. Applying Newton’s second law and torque balance equation, the lateral and yaw movement in the 2-DOF vehicle model can be described as follows [16]:

(1) $$\left\{ \begin{array}{c} ma_{y}=2F_{yf}+2F_{yr}\\ I_{z}\dot{\gamma }=2F_{yf}l_{f}-2F_{yr}l_{r} \end{array}\right.$$

where *m* and $I_{z}$ are the mass of vehicle and the moment about the vertical axis, respectively; $a_{y}$ represents the lateral acceleration at the center of gravity (CG), which can be described as $a_{y}={\dot{v}}_{y}+v_{x}\gamma =v_{x}\left(\dot{\beta }+\gamma \right)$ with the assumption of the constant longitudinal velocity [16]; $v_{x}$ and $v_{y}$ represent the longitudinal and lateral velocities at the CG; β and γ are the sideslip angle and yaw rate at the CG, respectively; $l_{f}$ and $l_{r}$ are the distances from the CG to the front and the rear axles; $F_{yf}$ and $F_{yr}$ are the lateral tire forces of the vehicle in the front wheel and rear wheel, respectively, which can be written as $F_{yf}=C_{f}\alpha_{f}$ and $F_{yr}=C_{r}\alpha_{r}$ with the assumption of small sideslip angle of tire; $C_{f}$ and $C_{r}$ stand for the cornering stiffness of the front and rear wheels respectively; $\alpha_{f}$ and $\alpha_{r}$ represent the front and rear wheel sideslip angles respectively, and they can be defined as:

(2) $$\left\{ \begin{array}{c} \alpha_{f}=\delta -\frac{v_{y}+l_{f}\gamma }{v_{x}}\\ \alpha_{r}=-\frac{v_{y}-l_{r}\gamma }{v_{x}} \end{array}\right.$$

where δ is the front wheel steering angle made from the driver.

Substituting Equation (2) into Equation (1), Equation (1) can be rewritten as:

(3) $$\left\{ \begin{array}{l} mv_{x}\left(\dot{\beta }+\gamma \right)=2C_{f}\left(\delta -\beta -l_{f}\gamma /v_{x}\right)+2C_{r}\left(-\beta +l_{r}\gamma /v_{x}\right)\\ I_{z}\dot{\gamma }=2C_{f}l_{f}\left(\delta -\beta -l_{f}\gamma /v_{x}\right)-2C_{r}l_{r}\left(-\beta +l_{r}\gamma /v_{x}\right) \end{array}\right..$$

By defining $x={\left[\beta ,\;\gamma \right]}^{T}$ as the state variables, Equation (3) can be represented in the form of:

(4) $$\dot{x}=Ax+B\delta ,$$

where *A* and *B* are the state variable matrix and control input matrix, respectively. These two matrices can be given as:

(5) $$A=\left[\begin{array}{cc} -\frac{2C_{f}+2C_{r}}{mv_{x}} & -1-\frac{2l_{f}C_{f}-2l_{r}C_{r}}{mv_{x}^{2}}\\ -\frac{2l_{f}C_{f}-2l_{r}C_{r}}{I_{z}} & -\frac{2l_{f}^{2}C_{f}+2l_{r}^{2}C_{r}}{I_{z}v_{x}} \end{array}\right],\;B=\left[\begin{array}{c} \frac{2C_{f}}{mv_{x}}\\ \frac{2l_{f}C_{f}}{I_{z}} \end{array}\right].$$

## 3. Proposed AFS System with the VGRS Actuator

**Figure 2:**
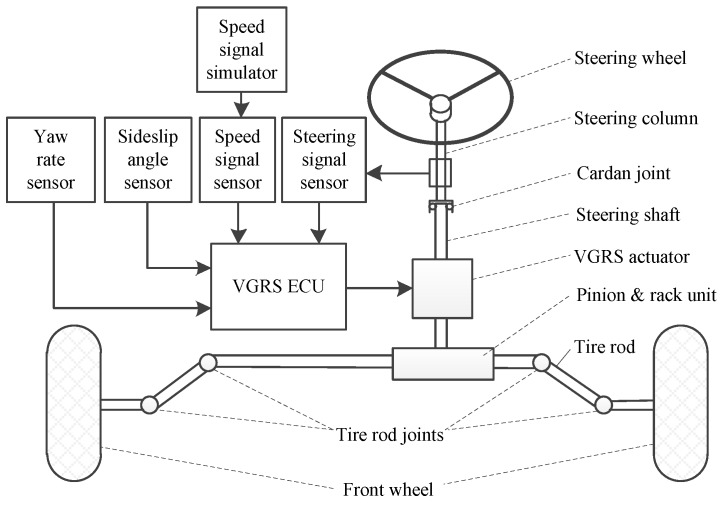
Construction of AFS system with the VGRS actuator.

Since the steering system is coupled with other vehicle parts and it is difficult to investigate in a real vehicle, a simplified model for the AFS system with the VGRS actuator is established to study the cornering dynamics. The AFS system consists of the VGRS actuator, signal sensors, speed signal, VGRS electronic control unit (ECU), steering wheel, steering column, cardan joint, steering shaft, pinion and rack unit, tire rods, tire rod joints, two front wheels, etc. Figure 2 shows the construction of the AFS system with the VGRS actuator. The signal sensors measure the real-time vehicle state, including the steering signal, vehicle speed, yaw rate, and sideslip angle. The VGRS actuator is used to produce an additional operating angle independent of the driver’s input, which is in accordance with the control command of the VGRS ECU. In addition, The VGRS ECU sends out a command signal according to the vehicle states, such as speed, yaw rate, and sideslip angle. The steering column is connected to the steering wheel and coupled to the steering shaft by a cardan joint. The VGRS actuator is located at the end of the steering shaft. Next, the pinion and rack unit linked with the output shaft of VGRS actuator turns the front wheels through the tire rod and rod joint.

**Figure 3:**
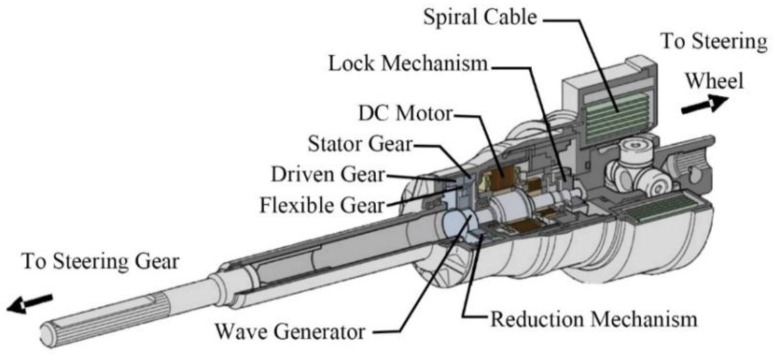
Construction of the VGRS actuator [17].

Regarding the VGRS actuator, it is composed of a DC motor, reduction mechanism, lock mechanism, housing, and output shaft, as described in Figure 3. The DC motor is mounted on the housing with high power output and less noise. It rotates either clockwise or counterclockwise depending on the command from the VGRS ECU and connects with the reduction mechanism, which includes four main components: stator gear, driven gear, flexible gear, and wave generator. The stator gear coupled to the housing has a rigid body and ring shape. The driven gear is parallel with the stator gear and connects to the output shaft. The flexible gear has the same teeth as the driven gear, but it is located inside the stator gear and driven gear, as well as the outer surface of the wave generator with a flexible metal body. In addition, the number of the teeth of the stator gear is more than that of the other kinds of gears in the VGRS actuator, thus forming a reduction mechanism to reduce the speed from the DC motor to the driven gear. For example, a 50:1 speed ratio is generated with 102 teeth for the stator gear and 100 teeth for both the flexible gear and driven gear. The wave generator coupled to the DC motor shaft transmits angular motion to the flexible gear. For the lock mechanism, it is located on the DC motor and mechanically locks the motor to avoid negative effects on steering operation when a failure occurs in the VGRS actuator. Upon failure, the housing and output shaft become united and the DC motor stops working. The housing covers all of the components, and the output shaft of the VGRS actuator is finally connected to the steering gear assembly.

**Figure 4:**
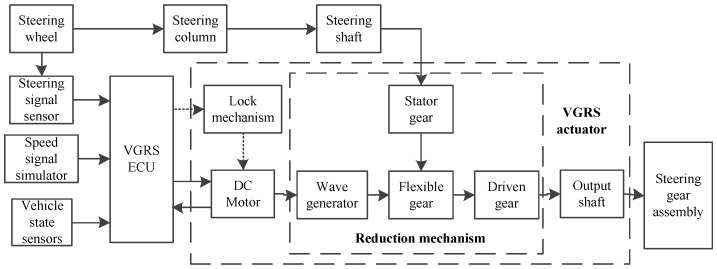
Workflow of AFS system with the VGRS actuator.

**Figure 5:**
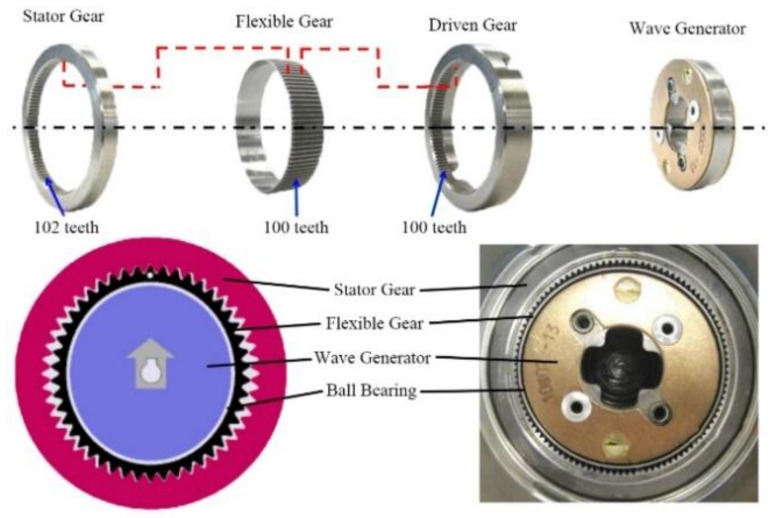
Structure of the reduction mechanism [17].

Figure 4 shows the work flow of the AFS with the VGRS actuator. A steering input angle is generated from the steering wheel and transmitted to the steering shaft via steering column, which is also measured by the steering angle sensor. The VGRS ECU receives the steering angle signal, speed signal, and other state signals, then it is processed by a controller and sends out the command to the DC motor to drive the wave generator. At the same time, the reduction mechanism rotates in accordance with the steering shaft. Figure 5 depicts the flexible gear couples to the outer surface of the wave generator and its teeth mesh with the internal gear of the stator. The flexible gear has 100 teeth while the stator has 102 teeth. There is a ball bearing between the flexible gear and the wave generator. The gear ratio of the wave generator and flexible gear is 50:1. This means that the flexible gear only rotates one round when the wave generator rotates 50 rounds. Then the output of the flexible gear combines with that of the wave generator before it is transferred to the driven gear. The steering gear finally obtains the turning angle from the driven gear.

**Figure 6:**
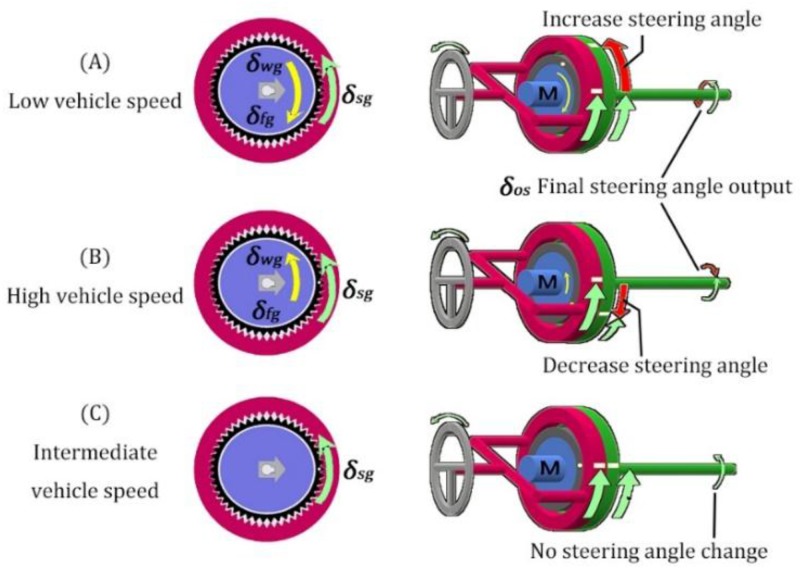
Three VGRS working modes [17].

Three working statuses of the VGRS are shown in Figure 6. While the vehicle speed is low, the wave generator rotates in the opposite direction to the steering wheel to drive the flexible gear and then to drive the driven gear as shown in Figure 6A. The flexible gear rotates one round every 50 rounds of the wave generator. Additionally, the driven gear rotates two teeth when the flexible gear rotates one round in the opposite direction. This means that the rotating direction of the driven gear is the same as that of the steering wheel. Thus, it can increase the steering angle in low vehicle speeds. On the contrary, while the vehicle speed is high, the wave generator rotates in the same direction as the steering wheel to drive the flexible gear. Then the driven gear rotates in the opposite direction to the steering wheel because the flexible gear rotates in the same direction of the steering wheel. This means that the final output steering angle is decreased as shown in Figure 6B. The lock mechanism locks when the vehicle speed is medium. Then the actuator works as a fixed one, where the gear ratio is 1:1. At this stage, the output angle is the same as the input angle as shown in Figure 6C.

**Figure 7:**
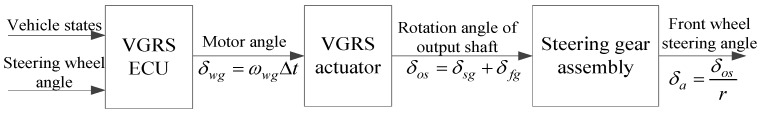
System block diagram of the VGRS actuator.

Figure 7 shows the system block diagram of VGRS actuator. To construct the VGRS model, the formula of gear ratio is firstly constructed:

(6) $$r_{gr}=\frac{\omega_{wg}}{\omega_{fg}}=\frac{n_{fg}}{n_{sg}-n_{fg}}=\frac{50}{1},$$

where $\omega_{wg}$ is the angular speed of the wave generator (i.e., the DC motor speed) and it is assumed to be a constant; $\omega_{fg}$ stands for the angular speed of the flexible gear; $n_{fg}$ and $n_{sg}$ are the numbers of the teeth of the flexible gear and stator gear, respectively.

Based on the working principle of VGRS, the driven gear rotates at the resultant angular speed, which can be calculated by summing the angular speed of the stator gear and flexible gear. Furthermore, the output shaft rotates at the same angular speed with that of the driven gear. So, the angular speed of output shaft can be written as:

(7) $$\omega_{os}=\omega_{sg}+\omega_{fg}=\omega_{sg}+\frac{\omega_{wg}}{r_{gr}},$$

where $\omega_{os}$ and $\omega_{sg}$ are the angular speed of the output shaft and the angular speed of the stator gear, respectively.

Since the rotation angle is proportional to the angular speed, the rotation angle of the output shaft can be represented as:

(8) $$\delta_{os}=\delta_{sg}+\delta_{fg}=\delta_{sg}+\frac{\delta_{wg}}{r_{gr}},$$

where $\delta_{sg}$ and $\delta_{fg}$ are the rotation angle of the stator gear and rotation angle of flexible gear, respectively; $\delta_{os}$ stands for the rotation angle of the output shaft which is equal to the steering wheel angle; and $\delta_{wg}$ is the rotation angle of the wave generator which can be calculated as:

(9) $$\delta_{wg}=\omega_{wg}\Delta t,$$

where Δt is the rotation time of wave generator which is the time delay between the controller output steering angle and the actual steering angle. When the wave generator rotates in the same direction as the stator gear, the rotation angle of the wave generator is positive. On the contrary, the rotation angle of output shaft decreases with a negative rotation angle of the wave generator when the wave generator rotates in the opposite direction with the stator gear. Additionally, the rotation angle of the output shaft is equal to the rotation angle of the stator gear when there is no action from the DC motor.

After transmitting via steering gear assembly, the actual front wheel steering angle can be represented as:

(10) $$\delta_{a}(t)=\delta_{a}=\frac{\delta_{os}}{r}=\frac{\delta_{sg}}{r}+\frac{\delta_{wg}}{r_{gr}r}=\delta +\frac{\delta_{wg}}{r_{gr}r}=\delta (t)+\frac{\omega_{wg}\Delta t}{r_{gr}r},$$

where *r* is the gear ratio of the steering gear assembly. Finally, the relationship between the controller output steering angle and the actual steering angle in time-domain can be expressed as:

(11) $$\delta_{c}(t)=\delta_{a}(t+\Delta t),$$

where $\delta_{c}\left(t\right)$ is the controller output steering angle.

## 4. Controller Design for the Proposed System

**Figure 8:**
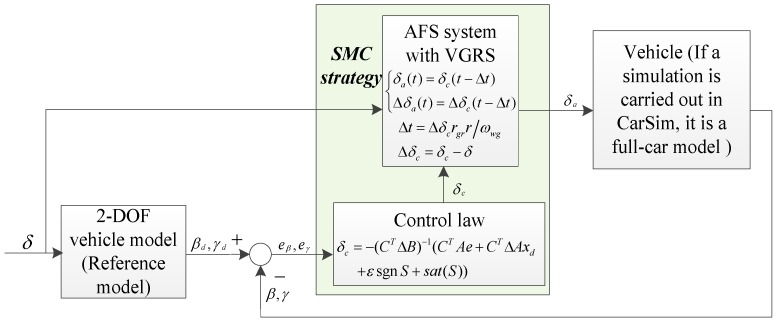
Control block diagram of the proposed system.

In this section, the controller for the proposed AFS system with the VGRS actuator is designed and the control block diagram is shown in Figure 8. The 2-DOF vehicle model is used to calculate the desired values of the sideslip angle and yaw rate by considering the steady steering and transient response. Moreover, the control law is deduced based on the 2-DOF vehicle model by defining the switching surface and selecting the reaching law. Based on the output steering angle from the control law and the front wheel steering angle from the driver, the actual wheel steering angle can be offered to the vehicle by the VGRS actuator. If a simulation is carried out in CarSim (Mechanical Simulation Corporation, Ann Arbor, MI, USA, a well-known commercial software which can provide accurate and realistic conditions close to the real car test), the vehicle is a full-car model. Subsequently, the actual values of sideslip angle and yaw rate can be obtained from the vehicle/full-car model. Then, by subtracting the actual values from the desired values, the errors of sideslip angle and yaw rate can be calculated. Their details are presented in the following subsections.

### 4.1. Errors of Sideslip Angle and Yaw Rate

In fact, the sideslip angle and yaw rate usually change during a maneuver. However, by applying control strategies, the sideslip angle and yaw rate are controlled to approximate their desired values. In this part, the desired values of the sideslip angle and yaw rate are defined, which means that the vehicle is assumed to run in the steady-state condition. When the vehicle works in a steady-state driving condition, the sideslip angle and yaw rate are expected to be constant, thus obtaining a good driving sense and cornering stability. In this state, the values of sideslip angle and yaw rate are calculated to obtain the desired value.

Considering the steady-state driving condition, the sideslip angle and yaw rate are kept constant, which indicates that the rate of them are all zero. Then the values of sideslip angle and yaw rate in steady-state can be obtained and these values are also regarded as desired values. Meanwhile, the desired sideslip angle and desired yaw rate are bounded to avoid the lateral force to reach its limitation in a large lateral acceleration. So, by integrating Equation (3) and assuming steady-state driving condition, the desired values of sideslip angle $\beta_{s}$ and yaw rate $\gamma_{s}$ are represented as:

(12) $$\beta_{s}=\left\{ \begin{array}{cc} \frac{\left(l_{r}-\frac{l_{f}mv_{x}^{2}}{2C_{r}\left(l_{f}+l_{r}\right)}\right)\delta }{l_{f}+l_{r}+\frac{\left(l_{r}C_{r}-l_{f}C_{f}\right)mv_{x}^{2}}{2C_{f}C_{r}\left(l_{f}+l_{r}\right)}}, & \beta_{s}\leq \arctan\left(0.02\mu g\right)\\ \arctan\left(0.02\mu g\right), & \beta_{s}>\arctan\left(0.02\mu g\right) \end{array}\right.$$

and:

(13) $$\gamma_{s}=\left\{ \begin{array}{cc} \frac{v_{x}\delta }{l_{f}+l_{r}+\frac{\left(l_{r}C_{r}-l_{f}C_{f}\right)mv_{x}^{2}}{2C_{f}C_{r}\left(l_{f}+l_{r}\right)}}, & \left|\gamma_{s}\right|\leq 0.85\frac{\mu g}{v_{x}}\\ 0.85\frac{\mu g}{v_{x}}\;\mathrm{sgn}\left(\gamma_{d}\right), & \left|\gamma_{s}\right|>0.85\frac{\mu g}{v_{x}} \end{array}\right..$$

However, the aforementioned desired values of sideslip angle and yaw rate are both under steady steering. Taking the characteristics of transient response, a first-order element with differential delay ($\tau_{\beta }$ for sideslip angle and $\tau_{\gamma }$ for yaw rate) is introduced [18]. Thus, the desired sideslip angle $\beta_{d}$ and yaw rate $\gamma_{d}$ in the bounded zone can be represented as:

(14) $$\left\{ \begin{array}{c} \beta_{d}=\frac{1}{1+\tau_{\beta }s}\cdot \beta_{s}=\frac{G_{\beta }\left(0\right)}{1+\tau_{\beta }s}\delta \\ \gamma_{d}=\frac{1}{1+\tau_{\gamma }s}\cdot \gamma_{s}=\frac{G_{\gamma }\left(0\right)}{1+\tau_{\gamma }s}\delta \end{array}\right..$$

where:

$$\left\{ \begin{array}{c} G_{\beta }\left(0\right)=\frac{l_{r}-\frac{l_{f}mv_{x}^{2}}{2C_{r}\left(l_{f}+l_{r}\right)}}{l_{f}+l_{r}+\frac{\left(l_{r}C_{r}-l_{f}C_{f}\right)mv_{x}^{2}}{2C_{f}C_{r}\left(l_{f}+l_{r}\right)}}\\ G_{\gamma }\left(0\right)=\frac{v_{x}}{l_{f}+l_{r}+\frac{\left(l_{r}C_{r}-l_{f}C_{f}\right)mv_{x}^{2}}{2C_{f}C_{r}\left(l_{f}+l_{r}\right)}} \end{array}\right..$$

Once the desired valued is obtained, the errors of sideslip angle and yaw rate can be represented as:

(15) $$\left\{ \begin{array}{c} e_{\beta }=\beta -\beta_{d}\\ e_{\gamma }=\gamma -\gamma_{d} \end{array}\right..$$

By defining $e={\left[e_{\beta },e_{\gamma }\right]}^{T}$ and $x_{d}={\left[\beta_{d},\gamma_{d}\right]}^{T}$, the errors between the actual value and desired value can be rewritten as:

(16) $$e=x-x_{d}.$$

Additionally, the desired values can be written in the form of state equation by taking the inverse Laplace transformation of Equation (14):

(17) $$\begin{array}{c} {\dot{x}}_{d}=A_{d}\cdot x_{d}+B_{d}\cdot \delta \\ \left\{ \begin{array}{c} A_{d}=\left[\begin{array}{cc} -1/\tau_{\beta } & 0\\ 0 & -1/\tau_{\gamma } \end{array}\right]\\ B_{d}={\left[\begin{array}{cc} G_{\beta }\left(0\right)/\tau_{\beta } & G_{\gamma }\left(0\right)/\tau_{\gamma } \end{array}\right]}^{T} \end{array}\right. \end{array}.$$

Then, the derivative of errors can be obtained:

(18) $$\begin{array}{c} \dot{e}=\dot{x}-{\dot{x}}_{d}=Ae+\Delta Ax_{d}+\Delta B\delta \\ \Delta A=A-A_{d}\\ \Delta B=B-B_{d} \end{array}.$$

### 4.2. SMC Strategy

In terms of the linear system described in Equation (18), the switching surface can be constructed as

(19) $$S=C^{T}e=ce_{\beta }+e_{\gamma }.$$

In the SMC strategy, the parameter *c* should satisfy the condition that (*p* + *c*) is Hurwitz polynomial. This means that the eigenvalue p of the polynomial *p* + *c* = 0 should be negative. In other words, the parameter c must be a positive number. In this work, the value of c is selected by a trial and error method. After determining the value of *c*, the switching surface is designed with the matrix of $C={\left[c\;1\right]}^{T}={\left[2\;1\right]}^{T}$. Thus, the switching surface is $S=2e_{\beta }+e_{\gamma }$.

Apart from the design of switching surface, the approaching motion is also a crucial process in enhancing the dynamic performance of the system. The approaching motion refers to the motion of system from the initial state to the sliding manifold. A general form of the reaching law can be expressed as $\dot{S}=-QsgnS-kh\left(S\right)$. According to the general form of the reaching law, there are three kinds of common reaching laws which consist of constant rate reaching, power rate reaching, and constant plus proportional rate reaching. The constant rate reaching is simple but difficult to control the approaching rate and chattering problems. The parameter tuning in power rate reaching is difficult to achieve a good dynamic performance. However, the constant plus proportional rate reaching behaves better in reaching time and lower chattering, as well as parameter tuning [19]. Thus, to improve the dynamic characteristics of approaching motion, the constant plus proportional rate reaching is selected:

(20) $$\dot{S}=-\varepsilon \mathrm{sgn}S-kS=C^{T}\dot{e}=C^{T}(Ae+\Delta Ax_{d}+\Delta B\delta ).$$

To further prevent chattering problems, the proportional item is replaced by sat(*S*) which is defined as:

(21) $$sat(S)=\left\{ \begin{array}{cc} 1, & S>0.01\\ kS, & \left|S\right|\leq 0.01\;\mathrm{and}\;k=1/0.01\\ -1, & S<-0.01 \end{array}\right.$$

It is noteworthy that the values of ±0.01 and *k* are selected by a trial and error method. Then the control law can be designed as:

(22) $$\delta_{c}=-{\left(C^{T}\Delta B\right)}^{-1}(C^{T}Ae+C^{T}\Delta Ax_{d}+\varepsilon \mathrm{sgn}S+sat(S)).$$

It can be easily observed that $\left|C^{T}\Delta B\right|\neq 0$, so the matrix $C^{T}\Delta B$ is non-singular and invertible. As $\delta_{c}$ is the overall front steering wheel angle from the controller, $\Delta \delta_{c}$ is the AFS operating angle from the controller (i.e., the additional steering angle acted on the front wheel) can be obtained by:

(23) $$\Delta \delta_{c}=\delta_{c}-\delta .$$

By referring to Equation (11), the actual steering angle $\delta_{a}$ and the actual AFS operating angle, $\Delta \delta_{a}\left(t\right),$ can be rewritten as:

(24) $$\left\{ \begin{array}{c} \delta_{a}(t)=\delta_{c}(t-\Delta t)\\ \Delta \delta_{a}(t)=\Delta \delta_{c}(t-\Delta t) \end{array}\right.$$

then:

(25) $$\delta_{a}(t)=\Delta \delta_{a}(t)+\delta (t-\Delta t).$$

After calculating the actual steering angle, the time delay Δt should be found. By combining Equation (10) and Equation (23), the rotation angle of the DC motor can be obtained by:

(26) $$\delta_{wg}=\Delta \delta_{c}r_{gr}r=\omega_{wg}\Delta t.$$

Hence, the time delay can be calculated by:

(27) $$\Delta t=\frac{\Delta \delta_{c}r_{gr}r}{\omega_{wg}}.$$

In order to guarantee the reachability, stability and existence conditions of a sliding mode motion, the stability analysis is necessary.

By considering the following Lyapunov candidate function:

(28) $$V=\frac{1}{2}s^{2}\geq 0,$$

its derivative can be written as:

(29) $$\dot{V}=S\dot{S}=-S\cdot \mathrm{sgn}S-kS\cdot sat(S)\leq 0,$$

Obviously, the derivative of the Lyapunov candidate function is always negative, except that *S* = 0. So, the stability of the proposed SMC strategy can be guaranteed.

## 5. Simulation

**Figure 9:**
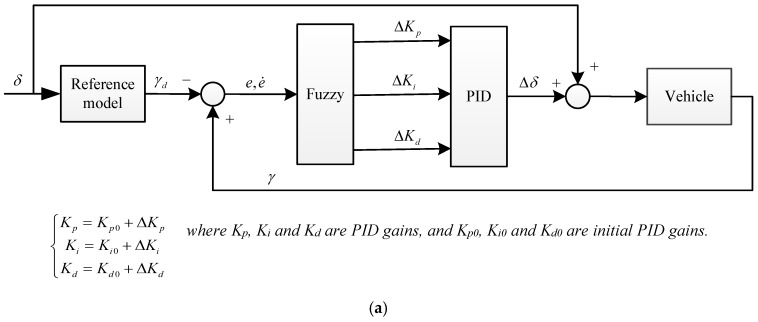

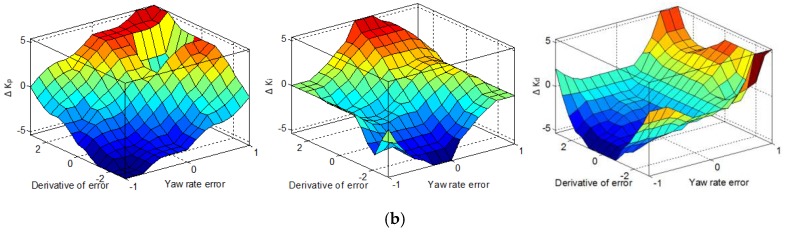
Design of fuzzy-PID strategy for AFS system: (**a**) flowchart; and (**b**) compositional rule of inference for the fuzzy controller.

Simulation is conducted in this section to test the proposed SMC control strategy in AFS system which is designed with the VGRS actuator. MATLAB (The MathWorks, Inc., Natick, MA, USA) and CarSim are utilized to perform the simulation based on the following discussion. In the simulation, a C-class hatchback vehicle, provided by the datasets of the CarSim, is employed. The main configuration of the C-class hatchback vehicle is described in Table 1. To examine the superiority of the proposed SMC strategy for concurrent sideslip angle and yaw rate control, a comparative case that only controls the yaw rate should be considered. In the open literature, the fuzzy-proportion-integration-differentiation (fuzzy-PID) strategy has been widely used for the AFS system with the advantage of robustness, simplification and quick response in comparison with other methods [20,21,22]. Considering the good performance described in recent literature, the fuzzy-PID strategy is also designed for the AFS with the VGRS actuator as a comparative case. For the fuzzy-PID strategy, only the yaw rate is controlled. The relevant flowchart and compositional rule of inference are shown in Figure 9. The parameters in the fuzzy-PID are set according to [21]. In the comparative study, the gear ratio of the steering gear is set as 16.5. The vehicle speed is assumed to be constant 80km/h (under the critical point) and two maneuvers are utilized to evaluate their performances. The first one is an open-loop steering maneuver for a Sine with Dwell test while the other one is a closed-loop steering maneuver for an accident avoidance test [23]. The simulation parameters are listed in Table 2. During the simulation, there is a time delay because of the motor speed and the gear ratio of the VGRS system.

### 5.1. Sine with Dwell Test

**Figure 10:**
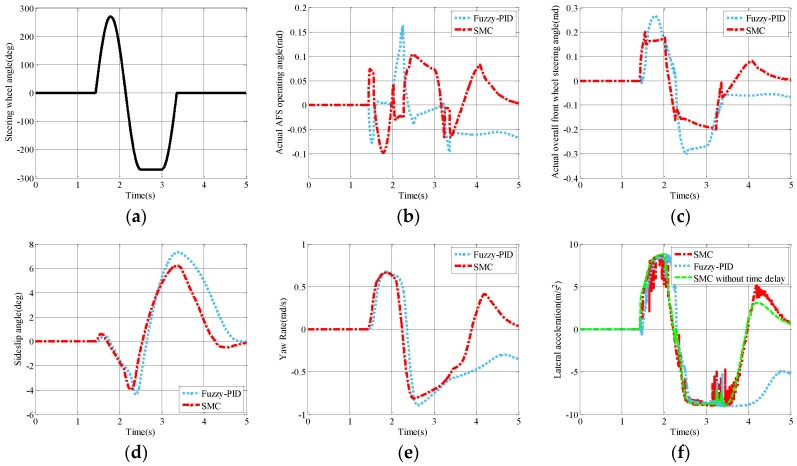
Simulation results of the Sine with Dwell test: (**a**) steering wheel angle; (**b**) actual AFS operating angle; (**c**) actual overall front wheel steering angle; (**d**) sideslip angle; (**e**) yaw rate; and (**f**) lateral acceleration.

Regarding the open-loop steering maneuver, an input of steering wheel angle from the driver (Sine with Dwell) is taken to conduct the test. The steering pattern includes a sine wave and a dwell time of 0.5 s, which conforms to the National Highway Traffic Safety Administration (NHTSA) standard, as shown in Figure 10a. The maximum steering angle is 270° and the frequency of the sine wave is 0.7 Hz [23]. With this input, the Sine with Dwell test is conducted and the simulation results are shown in Figure 10b–f.

Figure 10b,c illustrates the actual AFS operating angle and the actual overall front wheel steering angle respectively. Compared with the fuzzy-PID strategy, the actual AFS operating angle changes in a smaller range. Specifically, there exists a sudden change for the actual AFS operating angle under the fuzzy-PID strategy when the steering wheel angle changes from positive to negative, whereas the SMC can offer a relatively smooth change of the actual AFS operating angle. A sudden change of the operating angle will cause a rapid directional change of the DC motor output. This means that the proposed SMC strategy could provide better a lifetime of the VGRS actuator.

As for the evaluation indices, the sideslip angle, yaw rate, and lateral acceleration are selected to quantify the cornering stability, as shown in Figure 10e,f. By looking at the sideslip angle *β* of both control strategies shown in Figure 10d, the SMC strategy has smaller values in most cases and it is much faster in terms of reaching steady state. Obviously, instability also exists after a frequent steering under both SMC and fuzzy-PID strategies. In comparison with the SMC strategy, the yaw rate and lateral acceleration may lose control after the frequent steering under the fuzzy-PID strategy. Figure 10f shows that the time delay due to the rotation of the DC motor in the VGRS actuator leads to many jerks (the uneven region) in the curve of lateral acceleration in both fuzzy-PID and SMC strategies. Furthermore, the improvements in peak values and root mean square (RMS) values are also calculated in Table 3. The smaller the evaluation index, the better the performance of the system is. Under the SMC strategy, the peak values and the RMS values of sideslip angle, yaw rate, and lateral acceleration are all decreased compared with the fuzzy-PID strategy.

### 5.2. Accident Avoidance Test

**Figure 11:**
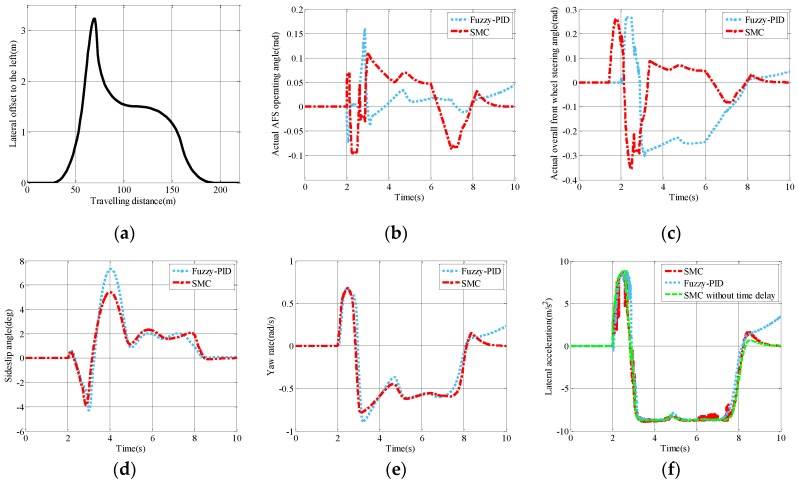
Simulation results of accident avoidance test: (**a**) Lateral offset to the left; (**b**) actual AFS operating angle; (**c**) actual overall front wheel steering angle; (**d**) sideslip angle; (**e**) yaw rate; and (**f**) lateral acceleration.

To examine the closed-loop steering maneuver, the accident avoidance test is conducted with a constant speed (80 km/h) to follow the given path as shown in Figure 11a. To avoid the accident, the driver has to turn the steering wheel urgently to drive the car to the left of the road. Then the car returns to the original lane slowly to ensure normal driving. As a result, the lane is changed to the left and then back to the right without using throttle or braking to control the vehicle. Considering the participation of the driver and frequent operation on the steering wheel, the accident avoidance test is one of the typical closed-loop steering maneuvers to test the cornering stability of the vehicle. Under the given driving condition, the simulation results are shown in Figure 11b–f.

From Figure 11b, even though the actual AFS operating angle is smoother in most cases under the fuzzy-PID strategy, a sudden change occurs when the car steers more to the left-hand side of the road. Apart from the actual AFS operating angle, the actual overall front wheel steering angle is shown in Figure 11c.

The sideslip angle, yaw rate, and lateral acceleration illustrated in Figure 11e,f and Table 4 are used to evaluate the cornering stability in the closed-loop steering maneuver. Generally speaking, the proposed SMC strategy provides good performance of sideslip angle, yaw rate, and lateral acceleration in comparison with the fuzzy-PID strategy. Table 4 evidently shows that the peak values of the three evaluation indices of the SMC strategy are lower than those of the fuzzy-PID strategy. Specifically, the peak values of sideslip angle, yaw rate and lateral acceleration under the SMC strategy outperform the fuzzy-PID strategy by 25.85%, 12.14%, and 0.87%, respectively. Additionally, even though the RMS values of the yaw rate and lateral acceleration only improve a little, the RMS value of the sideslip angle decreases by 21.73% under the SMC strategy. The reason may lie in that the fuzzy-PID strategy only controls one variable while the SMC strategy behaves well in controlling the two dependent objectives.

In the simulation of the two steering manoeuvers, a sudden change of actual AFS operating angle occurs under the fuzzy-PID strategy, while the actual AFS operating angle is smoother and changes in a smaller range under the SMC strategy. This implies that the SMC strategy behaves well in practice. Moreover, the simulation results also show that there are so many jerks in the curve of lateral acceleration in both fuzzy-PID and SMC strategies due to the time delay which is caused by the rotation of the DC motor in the VGRS actuator. To reduce the jerks and enhance the performance of cornering stability, the DC motor with high rotation speed should be considered in practice.

In addition, the improvement in the sideslip angle is larger as compared with the yaw rate. This is because only yaw rate is considered in the fuzzy-PID controller while both the sideslip angle and the yaw rate are considered in the proposed SMC strategy. Regarding the improvement in the yaw rate in Table 4, it is smaller than that of the yaw rate in Table 3. The reason may lie in that the maneuver for Table 4 is an accident avoidance test, in which a sharp steering angle is provided, as compared with the sine maneuver in Table 3. In such a serve/sharp steering condition, the proposed SMC strategy can still control the sideslip angle well. However, under a sharp steering, the lateral force of the tire may reach its saturation, resulting in failure in the control of the yaw rate. Therefore, little improvement in the yaw rate can be found in the accident avoidance test.

In short, the sideslip angle, yaw rate, and lateral acceleration can be decreased in both open-loop steering maneuver and closed-loop steering maneuver tests. Thus, the proposed SMC strategy performs better than the fuzzy-PID strategy in terms of cornering stability of vehicles.

## 6. Conclusions

In this work, an AFS system with the VGRS actuator is successfully designed and controlled using the SMC strategy so as to improve the cornering stability of vehicles. The main novelties of the paper are summarized as follows: In the design of the AFS system, different sensors are considered to measure the vehicle state, and the mathematical model of the VGRS is originally developed and considered. Moreover, in order to improve the cornering stability of vehicles, the two dependent objectives, sideslip angle and yaw rate, are originally considered together in the design of the SMC strategy. It is believed that the model of the proposed AFS system with the VGRS actuator will be helpful to the development of the intelligent steering system and improvement in vehicle cornering stability. Additionally, the proposed multi-objective SMC strategy can provide an alternative solution to researchers to solve the control problem with two dependent objectives.

## Figures and Tables

**Table 1:** Main configuration of a C-class hatchback vehicle.

Systems	Configuration
Internal engine model	125 kW engine
Internal transmission model	6-speed transmission
Internal differential	Viscous-Gear Ratio 4.1
Internal torque converter model	Torque converter for 125 kW engine
Tire	205/55 R16
Suspension type	Independent (Sprung mass: 1270 kg; front/rear unsprung mass: 71 kg for both sides)

**Table 2:** Parameters of the proposed AFS system with the VGRS actuator.

Parameter	Value	Unit	Parameter	Value	Unit
*m*	1412	kg	*l_r_*	1.458	m
*I_z_*	1536.7	kg·m^2^	*v_x_*	80	km/h
*C_f_*	49412	N/rad	*ω_wg_*	523.6	rad/s
*C_r_*	60174	N/rad	*r*	16.5	-
*l_f_*	1.016	m	*r_gr_*	50	-

**Table 3:** Improvements in peak values and RMS values.

	Peak Value			Root Mean Square Value		
	Fuzzy-PID	SMC	Improvement *	Fuzzy-PID	SMC	Improvement *
Sideslip angle (deg)	7.3350	6.2371	14.97%	3.3826	2.5911	23.40%
Yaw rate (rad/s)	0.8939	0.8127	9.08%	0.4701	0.4238	9.85%
Lateral acc. (m/s^2^)	9.0462	9.0289	0.19%	6.3378	5.3653	15.34%

* means the improvement of the SMC method relative to the fuzzy-PID method.

**Table 4:** Improvements in peak values and RMS values.

	Peak Value			Root Mean Square Value		
	Fuzzy-PID	SMC	Improvement *	Fuzzy-PID	SMC	Improvement *
Sideslip angle (deg)	7.3114	5.4213	25.85%	3.2171	2.5181	21.73%
Yaw rate (rad/s)	0.8914	0.7832	12.14%	0.4525	0.4501	0.53%
Lateral acc. (m/s^2^)	8.8230	8.7465	0.87%	6.1125	6.0970	0.25%

* means the improvement of the SMC method relative to the fuzzy-PID method.
